# Digital Smile Design-Guided Anterior Esthetic Rehabilitation With Lithium Disilicate Veneers: A Case Report

**DOI:** 10.7759/cureus.117691

**Published:** 2026-10-09

**Authors:** Padmapriya Puppala, Gaurang Mistry, Mishal De Souza, Swapnita Vaity, Nimrat Kaur Randhawa, Krsna Shetty

**Affiliations:** 1 Prosthodontics, DY Patil University School of Dentistry, Nerul, Navi Mumbai, IND

**Keywords:** ceramic veneers, digital smile design, esthetic dentistry, gingival zenith, lithium disilicate

## Abstract

Anterior esthetic rehabilitation requires careful coordination of tooth proportions, gingival architecture, shade correction, phonetics, and occlusal stability. This case report describes the management of a 27-year-old female patient who presented with dissatisfaction regarding her smile due to anterior spacing, tooth discoloration, asymmetric smile line, and gingival zenith discrepancy. A digital smile design protocol was used with exocad software (exocad GmbH, Darmstadt, Germany) to evaluate facial and dental reference lines, smile curve, tooth proportions, and planned gingival levels. A diagnostic wax-up and aesthetic pre-evaluative temporaries were fabricated to clinically validate the proposed outcome before definitive treatment. Gingival recontouring was performed using cautery, followed by a five-day healing period. Minimally invasive window veneer preparations with equigingival margins were then carried out from the maxillary right canine to the maxillary left canine. IPS e.max Press lithium disilicate veneers of shade A2 (Ivoclar Vivadent AG, Schaan, Liechtenstein) were fabricated and bonded using an adhesive resin cementation protocol. The final restorations achieved improved smile symmetry, closure of anterior spacing, correction of discoloration, and harmonious tooth proportions. At one-month follow-up, veneer retention, marginal adaptation, gingival health, phonetics, and patient satisfaction were satisfactory, with no sensitivity, chipping, or marginal staining.

## Introduction

Anterior esthetic rehabilitation requires careful coordination of facial features, lip dynamics, gingival architecture, tooth proportions, color, and occlusal function. Minor discrepancies in the maxillary anterior region, including spacing, altered incisal morphology, asymmetrical tooth dimensions, gingival zenith disharmony, and discoloration, can considerably influence smile perception and patient confidence [1,2]. These discrepancies become more noticeable in individuals with a high smile line because a greater portion of the maxillary anterior teeth and gingival margins is visible during smiling. Contemporary esthetic dentistry has therefore shifted from isolated correction toward a facially driven approach in which the final restorative outcome is planned in relation to the facial midline, lips, smile curve, and functional movements [3].

Digital smile design provides a structured method for evaluating these interrelated esthetic factors. Standardized extraoral and intraoral photographs, digital reference lines, and virtual tooth arrangements allow the clinician to visualize the proposed outcome before beginning irreversible treatment [4]. The digital design can subsequently be transferred to a three-dimensional printed model and reproduced clinically through a mock-up or preparation guide. This process improves communication among the patient, clinician, and dental laboratory while allowing potential discrepancies to be recognized during the planning stage [4,5]. Esthetic pre-evaluative temporaries further enable the proposed tooth form, incisal edge position, lip support, phonetics, and anterior guidance to be assessed before definitive preparation, thereby facilitating a more controlled and conservative treatment approach [6].

Ceramic laminate veneers are an established option for correcting anterior spacing, localized discoloration, and discrepancies in tooth form when adequate enamel is available for bonding [7]. Their clinical performance depends on appropriate case selection, enamel preservation, controlled preparation depth, reliable adhesive cementation, and careful occlusal evaluation [7,8]. Long-term evidence supports the durability of these restorations when appropriate clinical principles are followed. A systematic review estimated an overall cumulative survival rate of 89% over a median follow-up of nine years, while the corresponding estimated survival rate for glass-ceramic veneers was 94% [9]. Lithium disilicate is particularly suitable for anterior veneers because it combines favorable translucency and optical properties with adequate fracture resistance and compatibility with adhesive resin cementation.

Nevertheless, the extent of veneer treatment should correspond to the distribution and severity of the patient’s esthetic concerns. Localized defects may be managed with bleaching, direct composite restoration, orthodontic correction, or a single restoration, whereas multi-tooth rehabilitation should be considered only when spacing, tooth proportions, gingival relationships, and overall anterior symmetry require coordinated correction. Gingival recontouring may also be incorporated when uneven gingival zeniths remain visible and cannot be corrected through restorative contour alone. Treatment planning should therefore balance the desired esthetic outcome with preservation of sound tooth structure and consideration of less invasive alternatives. This case report describes an integrated digital and clinical workflow for planned anterior esthetic rehabilitation using gingival and ceramic restorative procedures.

## Case presentation

Patient information

A 27-year-old female patient reported to the Department of Prosthodontics with the chief complaint of spacing between the maxillary anterior teeth, discoloration of one anterior tooth, and an unsatisfactory appearance of her smile. She desired a natural-looking and definitive esthetic correction. Her medical and dental histories were non-contributory. There was no reported history of parafunctional habits, temporomandibular joint discomfort, or abnormal tooth wear.

Written informed consent was obtained from the patient for the proposed treatment and for the publication of anonymized clinical information and photographs. Institutional ethics committee approval was not required because this was a single-patient case report describing routine clinical care without an experimental intervention. The treatment and reporting were conducted in accordance with the ethical principles of the Declaration of Helsinki.

Clinical examination

Extraoral examination revealed an acceptable facial profile without any gross skeletal discrepancy. Evaluation during a full smile demonstrated a high smile line, which increased the visibility of the maxillary anterior gingival margins. The gingival zeniths were uneven, producing disharmony across the visible anterior segment. The purpose of subsequent gingival correction was to harmonize these gingival zeniths rather than alter the position of the upper lip or the overall smile line.

Intraoral examination revealed discoloration localized to the maxillary right central incisor (11), a midline diastema, and interproximal spacing involving the maxillary anterior teeth from 13 to 23. Asymmetrical tooth proportions and mild discrepancies in the incisal morphology of the anterior teeth were also observed. The clinical diagnosis was maxillary anterior spacing with localized discoloration of 11, asymmetrical tooth proportions, and gingival zenith discrepancy. The etiology of the discoloration could not be clearly established.

Periodontal examination showed satisfactory periodontal health without bleeding on probing. Occlusion was functionally acceptable, with canine guidance present during lateral excursive movements. Protrusive and lateral movements were evaluated, and no premature or unfavorable contacts were detected. No clinical signs of temporomandibular joint pathology, parafunctional activity, or abnormal wear facets were observed. Radiographic records were unavailable; therefore, treatment planning was based on the clinical examination, periodontal findings, esthetic analysis, and functional evaluation.

Digital smile design and diagnostic planning

Preoperative extraoral and intraoral photographs were obtained using standardized frontal, smiling, and retracted views. Digital planning was performed using exocad software version 3.3 (exocad GmbH, Darmstadt, Germany). The interpupillary line, facial midline, dental midline, smile curve, gingival levels, incisal edge positions, and tooth width-to-height proportions were evaluated.

A virtual diagnostic design was created for teeth 13, 12, 11, 21, 22, and 23 to assess the planned changes in tooth proportions, space distribution, incisal morphology, and gingival architecture. The completed exocad design was exported as a standard tessellation language file and used to fabricate a three-dimensional printed model. A soft splint was subsequently fabricated over the printed model to transfer the digitally planned gingival levels to the patient. A silicone putty index was prepared separately from the printed model for clinical mock-up fabrication, evaluation of tooth reduction, and provisionalization. Shade selection was performed before tooth dehydration, and A2 was selected as the definitive shade.

Treatment plan and clinical rationale

The treatment plan included gingival recontouring to harmonize the gingival zeniths, followed by ceramic laminate veneers from the maxillary right canine to the maxillary left canine. Although discoloration was confined to tooth 11, the patient’s concerns also included a midline diastema, interproximal spacing, asymmetrical tooth proportions, and uneven anterior tooth form across the canine-to-canine segment. A single veneer on 11 would have corrected only the localized discoloration and would not have addressed the spacing and proportional discrepancies involving the remaining anterior teeth.

Alternative treatment options, including bleaching, orthodontic space closure, direct composite restorations, treatment limited to tooth 11, and no treatment, were explained to the patient. Bleaching or a single veneer would not have corrected the generalized spacing and tooth-proportion discrepancies. Orthodontic treatment was recommended as a conservative option for space redistribution; however, the patient did not wish to undergo orthodontic therapy. Following discussion of the advantages, limitations, biological costs, and maintenance requirements of the available treatments, the patient selected canine-to-canine ceramic veneer rehabilitation.

Mock-up and esthetic pre-evaluative temporaries

The digitally planned design was transferred clinically using the silicone putty index prepared from the three-dimensional printed model. The index was loaded with provisional restorative material and positioned over the unprepared maxillary anterior teeth. After polymerization, the index was removed, and excess material was trimmed to obtain the clinical mock-up.

The mock-up was evaluated for tooth form, contour, incisal edge position, lip support, anterior symmetry, phonetics, and function. Occlusal contacts were examined during protrusive and lateral movements. The patient was instructed to use the esthetic pre-evaluative temporaries for seven days to assess comfort, appearance, phonetics, and function. The patient accepted the proposed design, and no major modifications were required before proceeding with definitive treatment.

Gingival recontouring

Because the patient had a high smile line, the uneven gingival zeniths were readily visible during smiling. The planned gingival levels were transferred intraorally using the soft splint fabricated over the three-dimensional printed model. The splint served as a clinical reference for marking the proposed gingival contours. Gingival recontouring was performed using electrocautery, and approximately 1 mm of marginal soft tissue was removed in the indicated areas (Figure 1). The procedure was limited to the soft tissue, and no osseous recontouring was performed.

**Figure 1:**
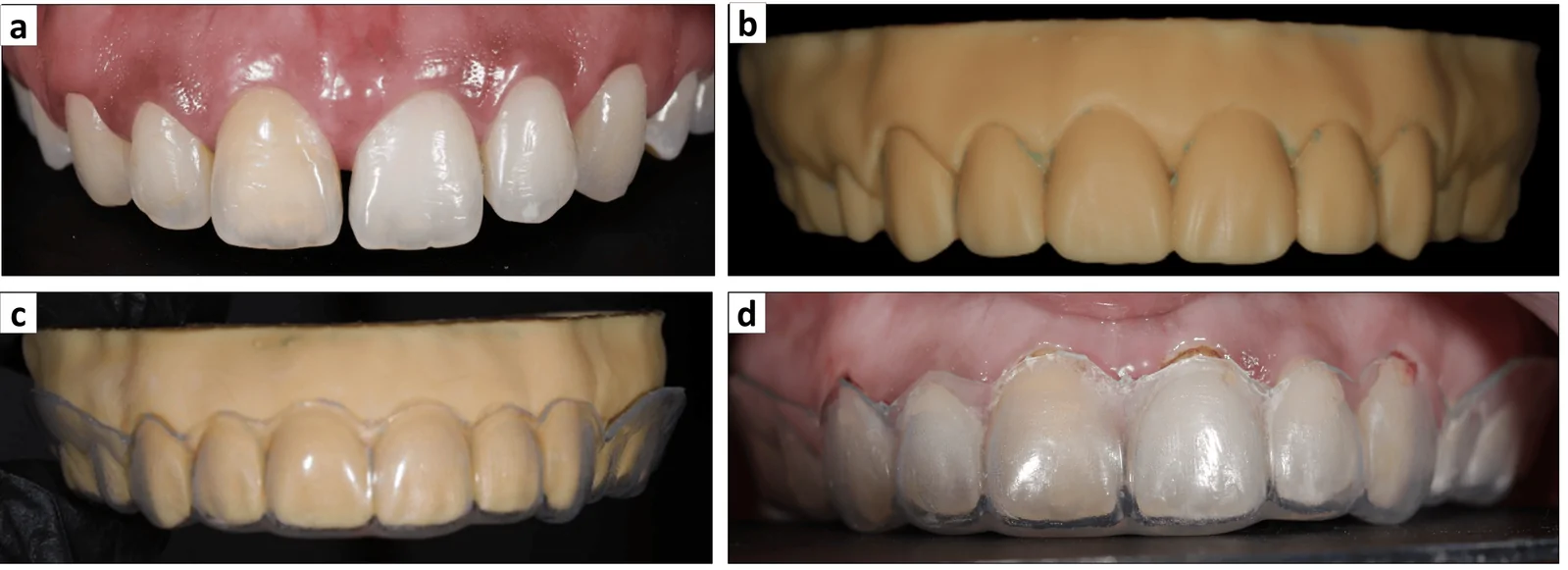
(a) Preoperative frontal view demonstrating the visible maxillary anterior segment; (b) digital mock-up; (c) digitally planned soft splint; and (d) splint-guided gingival recontouring performed using electrocautery.

The patient was recalled after five days. Clinical examination demonstrated satisfactory early healing without complications that would prevent further restorative treatment. Considering the limited amount of soft-tissue removal, absence of osseous intervention, and satisfactory clinical healing, definitive treatment was continued. The gingival contours remained satisfactory at the subsequent three-month evaluation.

Tooth preparation and final impression

Tooth preparation was performed through the approved mock-up using a veneer preparation protocol based on the technique described by Gürel [10]. Depth-orientation grooves were prepared through the mock-up to control the amount and distribution of facial reduction. The preparation depth was approximately 0.3 mm in the cervical third, 0.5 mm in the middle third, and 0.7 mm in the incisal third.

A window preparation design was used. The incisal edges were completely preserved, and the incisal termination of each preparation was positioned approximately 1 mm short of the incisal edge. Equigingival cervical margins were prepared to support esthetic integration while facilitating access for finishing, impression making, and maintenance of periodontal health.

The preparations were refined using rounded tapered fine diamond burs, and sharp internal line angles were smoothened. A silicone putty reduction index derived from the three-dimensional printed model was used to verify the available restorative space and prevent unnecessary tooth reduction. Gingival displacement was achieved using retraction cords. A two-stage final impression was made using heavy-body and light-body addition polyvinyl siloxane impression materials. The completed impression was examined for accurate reproduction of the prepared margins and then sent to the dental laboratory.

Provisionalization and laboratory phase

Provisional restorations were fabricated using the silicone putty index and retained through spot bonding. The provisionals were examined for marginal adaptation, tooth form, esthetics, phonetics, patient comfort, and occlusal contacts.

The definitive veneers were fabricated using IPS e.max Press lithium disilicate ceramic (Ivoclar Vivadent AG, Schaan, Liechtenstein). The approximate ceramic thickness was 1 mm, with variations according to the amount of space closure and the planned final contours. Shade A2 was used for the definitive restorations. The veneers were fabricated to close the anterior spaces, mask the localized discoloration of 11, improve tooth proportions, and establish a symmetrical canine-to-canine anterior arrangement. The preparation and provisionalization sequence is presented in Figure 2.

**Figure 2:**
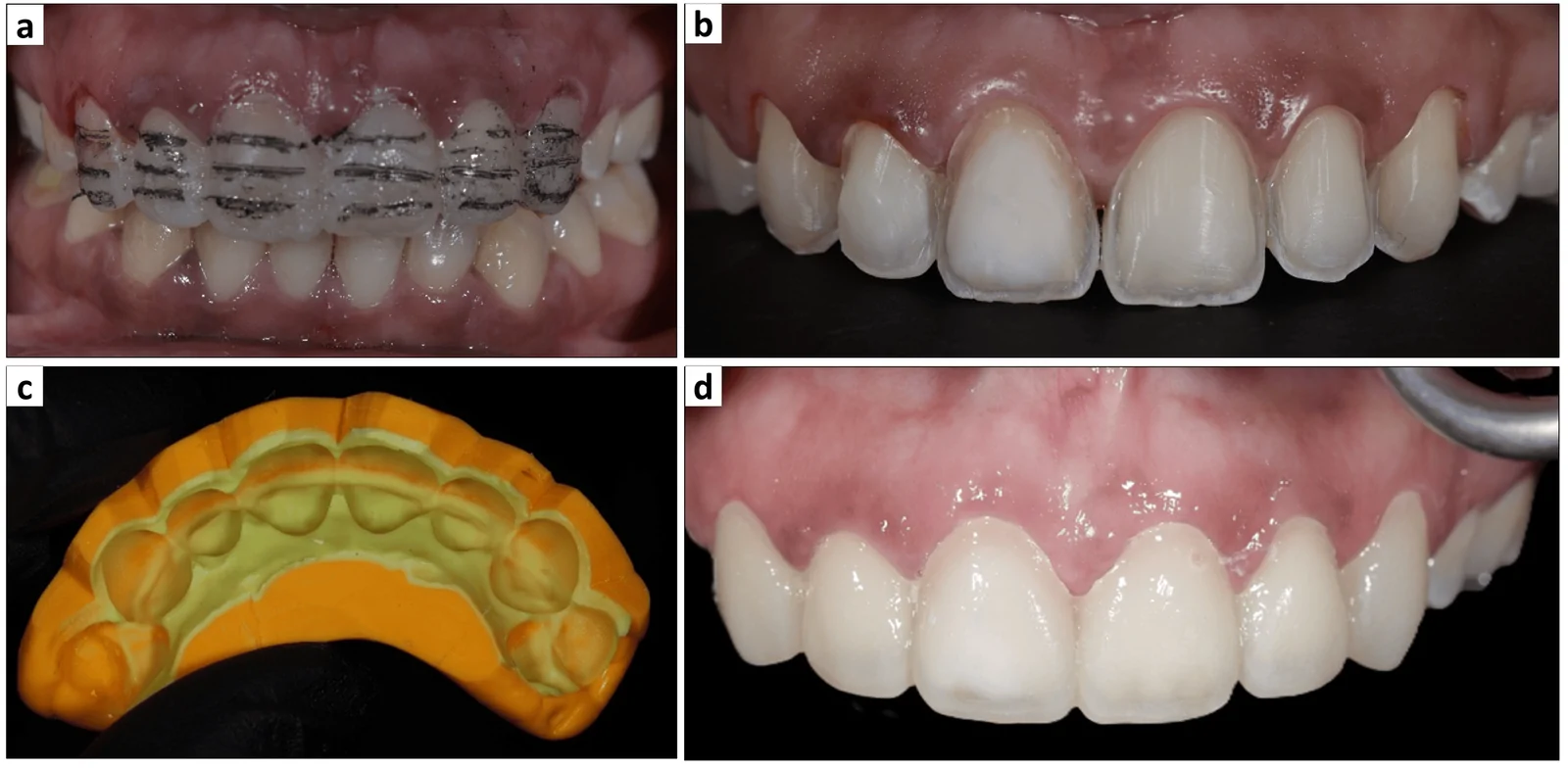
(a) Depth-orientation grooves prepared through the approved mock-up; (b) completed window veneer preparations with preservation of the incisal edges; (c) silicone putty index used for temporization; and (d) intraoral view of the provisional restorations.

Try-in and cementation

The patient was recalled for veneer try-in and definitive cementation. The provisional restorations were removed, and the prepared tooth surfaces were cleaned. Each veneer was individually examined for cervical adaptation, marginal fit, proximal contacts, contour, and shade. The restorations were then evaluated collectively to confirm the midline relationship, space closure, tooth proportions, and integration across the maxillary anterior segment. Try-in paste was used to assess the final optical appearance, and the patient approved the proposed result (Figure 3).

**Figure 3:**
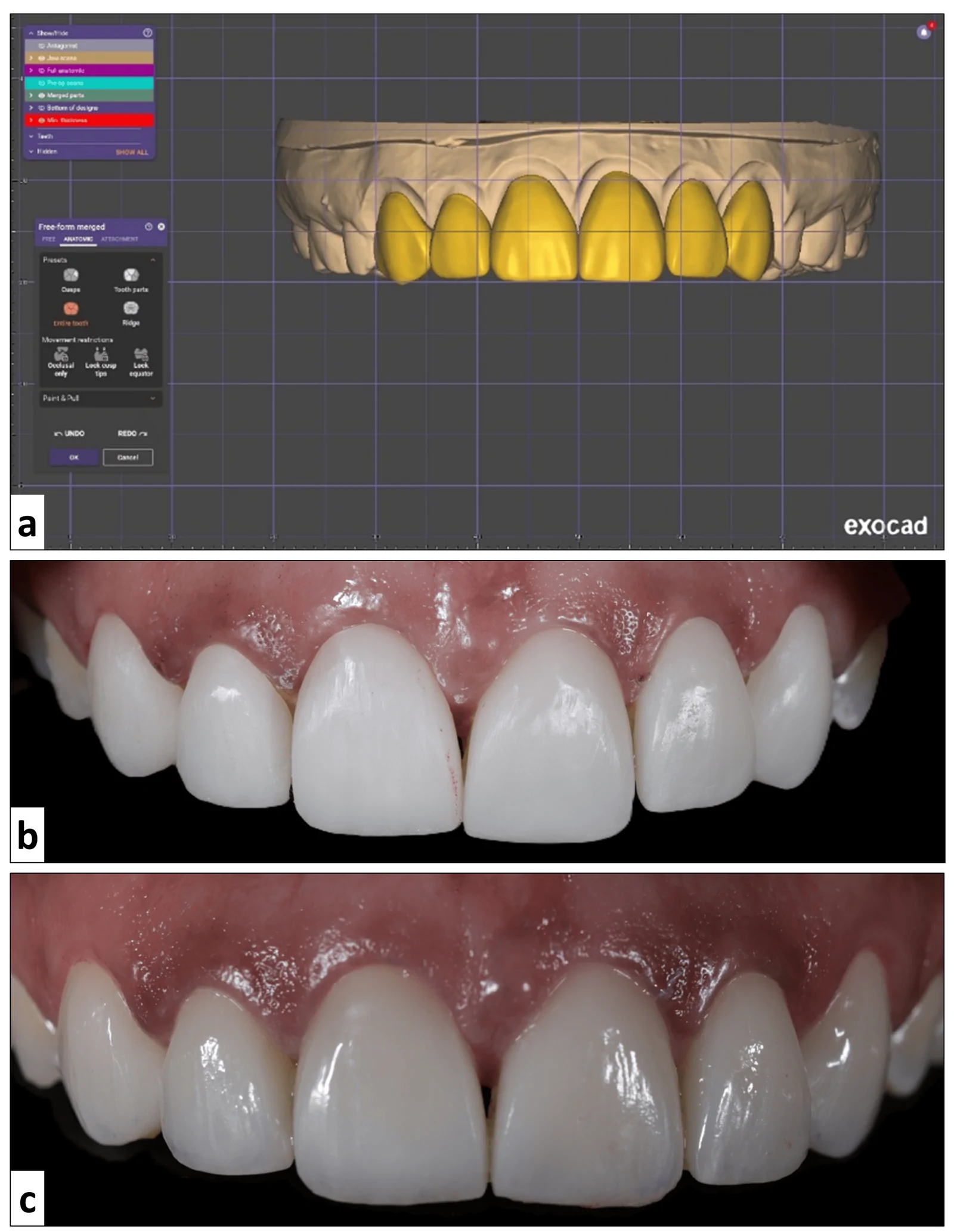
(a) Analysis of the definitive restorations using exocad software; (b) bisque try-in; and (c) completed lithium disilicate veneers. exocad GmbH, Darmstadt, Germany.

Isolation was achieved using cotton rolls and high-volume suction, while the adjacent teeth were protected with polytetrafluoroethylene tape. The internal ceramic surfaces were etched with 9.5% hydrofluoric acid for 20 seconds, rinsed thoroughly, and dried. A silane coupling agent was then applied according to the manufacturer’s instructions.

The prepared enamel surfaces were etched with 37% orthophosphoric acid gel (Restorite Etching Gel, Prime Dental Products Pvt. Ltd., Thane, Maharashtra, India) for 15 seconds. The surfaces were thoroughly rinsed and dried, following which the adhesive agent was applied and light-polymerized. The veneers were cemented using transparent-shade Variolink N resin cement (Ivoclar Vivadent AG, Schaan, Liechtenstein).

Each restoration was initially tack-cured for approximately three seconds to facilitate the removal of excess resin cement. Final polymerization was performed for 30 seconds from each surface using a Mini S light-emitting diode curing unit with an output intensity of 1000-1200 mW/cm² (Guilin Woodpecker Medical Instrument Co., Ltd., Guilin, China). Residual cement was removed from the cervical and interproximal areas using appropriate hand instruments and dental floss. The restoration margins were refined with fine diamond burs and sequentially polished using polishing discs.

After cementation, the occlusion was examined in centric relation, protrusive movement, and lateral excursions. Canine guidance was maintained, and no unfavorable contacts were detected on the veneers. Final marginal finishing and polishing were completed.

Postoperative outcome and follow-up

The definitive restorations achieved closure of the maxillary anterior spaces, masking of the localized discoloration of 11, and improved symmetry of the anterior tooth proportions. The gingival zeniths appeared more harmonious across the maxillary anterior segment. The high smile line itself remained unchanged because modification of the upper lip position was not an objective of treatment. Marginal adaptation and gingival response were clinically satisfactory, phonetics remained unaffected, and the patient expressed satisfaction with the immediate esthetic and functional outcome (Figure 4).

**Figure 4:**
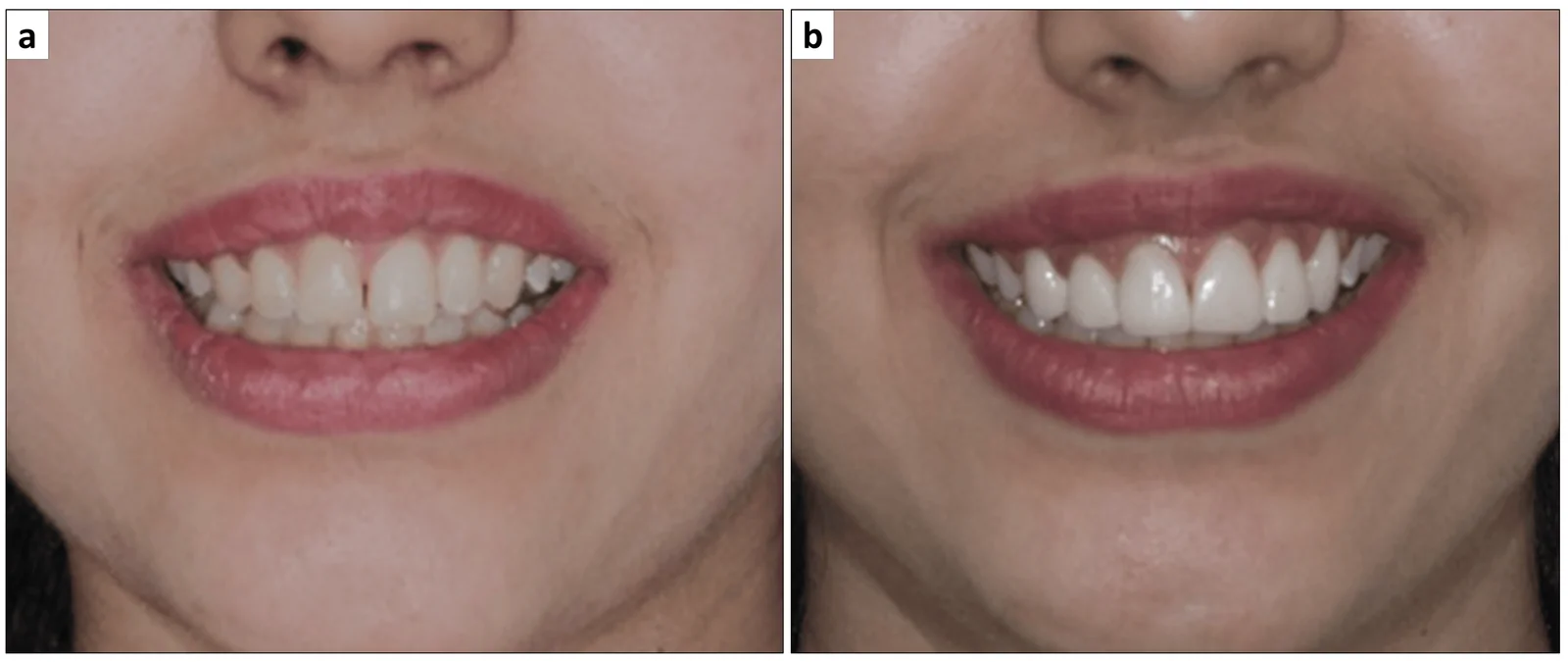
Comparison of the (a) preoperative and (b) postoperative smile

At the three-month follow-up, all six veneers remained retained and clinically satisfactory. No sensitivity, chipping, debonding, or marginal staining was observed. Gingival health was satisfactory, and the corrected gingival zeniths remained stable without clinically evident soft-tissue rebound. The patient continued to report satisfaction with the appearance and function of the restorations. Oral hygiene and maintenance instructions were reinforced, and periodic clinical follow-up was advised.

## Discussion

The present case demonstrates a digitally planned approach for managing multiple esthetic discrepancies involving the maxillary anterior region. The principal clinical challenge was not limited to masking tooth discoloration but included closure of anterior spacing, correction of asymmetrical tooth proportions, and harmonization of uneven gingival zeniths. Digital smile design can improve communication, patient involvement, and the transfer of esthetic planning to definitive treatment [11]. In the present case, it allowed the facial midline, lip position, smile curve, anterior tooth display, and gingival relationships to be evaluated collectively before treatment.

The conversion of the exocad design into a standard tessellation language file and subsequent fabrication of a three-dimensional printed model enabled the virtual plan to be transferred clinically. The model was used to produce a soft splint for transferring the planned gingival levels and a separate silicone putty index for the mock-up and provisional restorations. Digitally planned mock-ups allow potential esthetic and functional discrepancies to be recognized before irreversible treatment is initiated [12]. The seven-day use of esthetic pre-evaluative temporaries enabled the planned tooth contours, incisal positions, phonetics, lip support, and occlusal relationships to be assessed clinically.

An important consideration was the decision to provide veneers from 13 to 23 when discoloration was localized to tooth 11. The extent of treatment was not determined by the discoloration alone. The patient also presented with a midline diastema, interproximal spacing, asymmetrical anterior tooth proportions, and uneven tooth form across the canine-to-canine segment. Bleaching or a single veneer on 11 could have improved the localized color discrepancy but would not have corrected these multi-tooth problems. Orthodontic space closure was discussed as a conservative option, but the patient did not wish to undergo orthodontic treatment. Direct composite restorations and no treatment were also considered. Following an explanation of the advantages, limitations, biological cost, and maintenance requirements of each option, coordinated ceramic rehabilitation was selected to distribute space closure and establish more harmonious anterior proportions. Thus, the six-veneer approach should not be interpreted as a recommendation for treating an isolated discolored tooth.

Gingival recontouring was included because the patient had a high smile line that exposed the uneven gingival zeniths during smiling. Esthetic gingival procedures should be restoratively planned and should respect the periodontal and restorative landmarks necessary for stable gingival margins [13]. The objective in this case was to harmonize the gingival zeniths rather than alter the upper lip position or overall smile line. Approximately 1 mm of marginal soft tissue was removed using electrocautery without osseous recontouring. Although a longer interval is generally preferred for complete gingival maturation, treatment proceeded after five days because tissue removal was limited, no bone surgery was performed, and early healing was clinically satisfactory. The corrected gingival contours remained stable at three months; however, this case-specific timing should not be regarded as a general recommendation, particularly when more extensive soft-tissue or osseous correction is required.

The preparation depths were approximately 0.3 mm cervically, 0.5 mm in the middle third, and 0.7 mm in the incisal third. These graduated depths corresponded to the variation in enamel thickness and the restorative space required across the facial surface. Digitally guided preparation and the use of reduction indices can improve control over the amount of tooth reduction and help preserve the enamel substrate [14]. A window preparation was performed because the natural incisal edge was completely preserved and the incisal finish line terminated approximately 1 mm short of the edge. Favorable clinical outcomes have been reported for minimally invasive ceramic veneers when adequate enamel is preserved, and case selection is appropriate [15]. In the present case, depth grooves prepared through the approved mock-up and verification with a silicone reduction index helped prevent unnecessary tooth reduction.

Lithium disilicate was selected because it provides a useful combination of optical characteristics and mechanical strength for adhesively bonded anterior restorations. The approximately 1-mm ceramic thickness provided sufficient restorative volume for closure of the anterior spaces and masking of the localized discoloration of 11. The final optical appearance of lithium disilicate veneers is influenced by the type and thickness of the ceramic, underlying tooth color, and resin cement shade [16]. Shade selection was therefore performed before tooth dehydration, and try-in paste was used to assess the appearance before definitive cementation. These steps helped achieve a consistent transition between the treated teeth and the surrounding dentition.

Clinical longevity was another consideration during material selection and treatment planning. Systematic evidence supports favorable survival of ceramic laminate veneers, including lithium disilicate restorations, while identifying fracture, chipping, debonding, marginal discoloration, and biological complications as important outcomes requiring continued evaluation [17]. The absence of these complications at three months was encouraging but represented only an early clinical observation. It cannot be interpreted as evidence of long-term treatment survival in an individual patient.

The bonding procedure included hydrofluoric acid etching and silanization of the ceramic surfaces, followed by enamel conditioning with 37% orthophosphoric acid for 15 seconds. Appropriate ceramic surface treatment, tooth conditioning, resin cement selection, and moisture control are important for achieving reliable adhesion with lithium disilicate veneers [18]. Transparent-shade resin cement was used, and each restoration was initially tack-cured to facilitate excess cement removal. Final polymerization was performed for 30 seconds from each surface using an LED curing unit with an output intensity of 1000-1200 mW/cm². Cotton-roll isolation, high-volume suction, interproximal cleaning, and careful marginal finishing were used to reduce the likelihood of adhesive and periodontal complications.

Functional evaluation was performed before and after cementation. Canine guidance was maintained, and no unfavorable contacts were detected during protrusive or lateral movements. The absence of parafunctional habits, abnormal wear facets, and heavy contacts supported the selected restorative approach. Occlusal assessment remains clinically important because veneer complications may result from the combined effects of preparation design, bonding substrate, ceramic thickness, adhesive technique, and functional loading.

The principal limitation of this report is the three-month follow-up period. Although all veneers remained retained and no sensitivity, chipping, debonding, marginal staining, gingival inflammation, or soft-tissue rebound was observed, longer follow-up is required to evaluate ceramic integrity, color stability, adhesive durability, and periodontal response. Additional limitations include the absence of radiographic records, lack of a measured value for gingival display, and reliance on the patient’s reported satisfaction rather than a validated esthetic assessment instrument. Furthermore, the findings of a single case cannot be generalized to all patients with anterior spacing or localized discoloration. Continued clinical evaluation is therefore required before any conclusion regarding long-term treatment success can be made.

## Conclusions

Digital smile design, a three-dimensional printed model, mock-up validation, gingival recontouring, and conservatively prepared lithium disilicate veneers enabled coordinated correction of anterior spacing, localized discoloration, asymmetrical tooth proportions, and uneven gingival zeniths. At three months, the veneers remained clinically satisfactory without sensitivity, chipping, debonding, marginal staining, or gingival complications, and the patient reported good satisfaction. However, continued follow-up is necessary before conclusions regarding long-term survival and periodontal stability can be made.
